# Association of Coffee, Decaffeinated Coffee and Caffeine Intake from Coffee with Cognitive Performance in Older Adults: National Health and Nutrition Examination Survey (NHANES) 2011–2014.

**DOI:** 10.3390/nu12030840

**Published:** 2020-03-20

**Authors:** Xue Dong, Shiru Li, Jing Sun, Yan Li, Dongfeng Zhang

**Affiliations:** Department of Epidemiology and Health Statistics, The School of Public Health of Qingdao University, No. 308 Ningxia Road, Qingdao 266021, China; dongxue199411@126.com (X.D.);

**Keywords:** cognitive performance, decaffeinated coffee, caffeine intake from coffee, dose–response

## Abstract

The aim of this study was to examine the association of coffee, caffeinated coffee, decaffeinated coffee and caffeine intake from coffee with cognitive performance in older adults. we used data from the National Health and Nutrition Examination Survey (NHANES) 2011–2014. Coffee and caffeine intake were obtained through two 24-hour dietary recalls. Cognitive performance was evaluated by the Consortium to Establish a Registry for Alzheimer’s Disease (CERAD) test, Animal Fluency test and Digit Symbol Substitution Test (DSST). Binary logistic regression and restricted cubic spline models were applied to evaluate the association of coffee and caffeine intake with cognitive performance. A total of 2513 participants aged 60 years or older were included. In the fully adjusted model, compared to those reporting no coffee consumption, those who reported 266.4–495 (g/day) had a multivariate adjusted odd ratio (OR) with 95% confidence interval (CI) of 0.56(0.35–0.89) for DSST test score, compared to those reporting no caffeinated coffee consumption, those who reported ≥384.8 (g/day) had a multivariate-adjusted OR (95% CI) of 0.68(0.48–0.97) for DSST test score, compared to the lowest quartile of caffeine intake from coffee, the multivariate adjusted OR (95% CI) of the quartile (Q) three was 0.62(0.38–0.98) for the CERAD test score. L-shaped associations were apparent for coffee, caffeinated coffee and caffeine from coffee with the DSST test score and CERAD test score. No significant association was observed between decaffeinated coffee and different dimensions of cognitive performance. Our study suggests that coffee, caffeinated coffee and caffeine from coffee were associated with cognitive performance, while decaffeinated coffee was not associated with cognitive performance.

## 1. Introduction

Coffee is the leading beverage after water worldwide, and its trade exceeds US$10 billion [1]. Caffeine is present in many dietary sources consumed around the world, such as in coffee, tea, candy bars and cocoa beverages. The amount of caffeine ranges quite widely between these various foods, with coffee representing a major source of intake (71–220 mg caffeine/150 ml) [2,3,4]. Coffee has been shown to exert beneficial effects toward human health, including cardiovascular health, several types of cancer and neurodegenerative diseases [5], due to prevailing mechanisms such as inhibition of oxidative stress, regulation of DNA repair, phase II enzymatic activity, apoptosis and inflammation [6]. However, epidemiological evidence has shown that pregnant women and their offspring might be subjected to detrimental effects of caffeinated coffee [5].

As life expectancy increases, age-related cognitive decline can be a major health challenge for the elderly population [7], cognitive health has become an important public health issue for America’s aging population [8]. The process from cognitive decline to dementia is continuous and irreversible, and there is no effective treatment for dementia so far, the therapeutic value of drugs currently used is limited. Thus, developing measures to reduce risk for low cognitive performance as well as treatments of diagnosed dementia occupy a high priority in society. 

A number of epidemiological studies have demonstrated an association between higher coffee consumption and better cognitive performance [9,10,11,12,13]. Cognitive benefits from coffee consumption were typically attributed to caffeine. Caffeine is an antagonist of A1 and A2A adenosine receptors in the central nervous system and is known to have positive effects on attention, arousal, mood and vigilance [14,15,16]. Epidemiological studies have reported that caffeine was associated with cognitive impairment [17,18], suggesting that caffeine had a protective effect on cognitive performance. Furthermore, some studies of the association between caffeine and cognitive performance have also noted differential associations by gender, and the results were inconsistent [19,20,21,22]. However, few studies have explored the associations of decaffeinated coffee and caffeine intake from coffee with cognitive performance, and the results were inconsistent. Some studies have indicated that decaffeinated coffee might bring about some improvements to cognitive performance [23,24], while other studies have found that there was no significant association between decaffeinated coffee and cognitive function [25,26]. Moreover, some studies have suggested that caffeine from coffee was associated with cognitive performance [19,27], while other studies have reported null associations [28].

Therefore, we analyzed a nationally representative sample of older adults aged 60 years or older from the National Health and Nutrition Examination Survey (NHANES) to investigate the associations of total coffee, decaffeinated coffee, caffeinated coffee and caffeine intake from coffee with cognitive performance.

## 2. Materials and Methods 

### 2.1. Data Collection and Study Population

The National Health and Nutrition Examination Survey (NHANES) is a two-year-cycle cross-sectional survey conducted by the Centers for Disease Control and Prevention (CDC) of America, which aims to evaluate the health and nutritional status of the U.S. population [29]. Representative samples of the non-institutional U.S. population were selected by a complex stratified, multistage sampling design. Participants first took part in an interview at home and then carried out the health examination in a mobile examination center (MEC) [30]. The NHANES protocols were approved by the National Center for Health Statistics Ethics Review Board of the U.S. CDC, and written informed consent from all the participants was provided during the survey.

**Figure 1:**
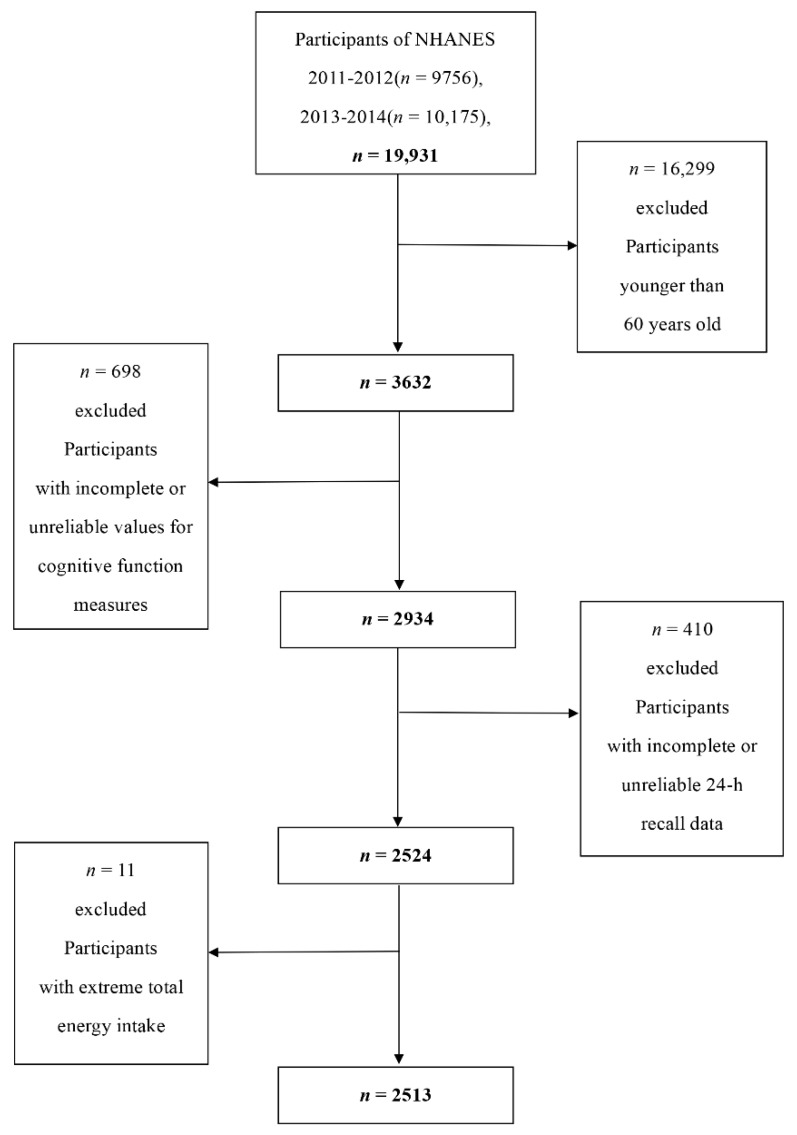
Flow chart of the screening process for the selection of eligible participants.

Two cycles (2011–2012, 2013–2014), with information on coffee and caffeine intake from coffee as well as cognitive function measures, were combined and used in the analysis. A total of 19,931 individuals participated in the NHANES from 2011 to 2014, and our analyses were limited to 3632 individuals aged 60 years or older. Among them, we excluded participants with incomplete cognitive tests or with unreliable values for the three cognitive function measures (n = 698), and incomplete or unreliable 24-hour recall data (n = 410). Individuals who had extreme total energy intakes of <500 or >5000 kcal/day for women, and <500 or >8000 kcal/day for men (n = 11) were further omitted. After exclusions, this study contained a total of 2513 participants aged 60 years or older (1214 men and 1299 women) (Figure 1). 

### 2.2. Cognitive Performance Assessment

A series of assessments for cognitive performance in the NHANES were used in the 2011–2014 survey, and cognitive tests were performed among participants aged 60 years or older [31]. Cognitive performance was assessed in a Mobile Examination Center (MEC) and was evaluated by the Consortium to Establish a Registry for Alzheimer’s Disease (CERAD) Word Learning sub-test, the Animal Fluency test and the Digit Symbol Substitution Test (DSST). These tests, which have been used in large screenings, epidemiological and clinical studies [32,33,34,35,36,37], evaluate working memory, language, processing speed and executive functioning in older adults. Participants were asked for consent to audio-record the administration for quality control purposes. Two interviewers transcribed responses from the audio recordings and scored the CERAD test, the Animal Fluency test and the DSST for interviews in English and Spanish. Transcription and scoring were usually done on the same day. Tests conducted in other languages were transcribed verbatim and scored by consultants in those languages later. If necessary, inconsistent scores were adjudicated by a third party. Approximately 10% of recorded interviews were independently reviewed during data collection [38].

The CERAD test consisted of three consecutive learning trials as well as a delayed recall, which were designed to assess immediate and delayed learning ability for new verbal information. In the learning trials, participants were organized to read aloud 10 unrelated words when they were presented one at a time. Immediately following the introduction of the words, participants recalled as many words as possible. The delayed word recall was completed after the Animal Fluency and DSST tests. The score on each trial ranged from 0 to 10, and the total score of the CERAD test was the sum of three learning trials and a delayed recall trial. As a component of executive function, the Animal Fluency test examined categorical verbal fluency, participants were called upon to name as many animals as possible in one minute. The score was the sum of the number of correct answers. The DSST, a performance module from the Wechsler Adult Intelligence Scale, was used to assess processing speed, sustained attention and working memory. The exercise was performed using a piece of paper with a key at the top pairing numbers with nine symbols. Participants had two minutes to copy the corresponding symbols from the 133 boxes that held adjacent numbers. The score, ranging from 0 to 133, was the sum for the number of correct matches. 

Currently, there is no gold standard of cutoff point for the CERAD, Animal Fluency and DSST tests to identify low cognitive performance. Therefore, we used the 25th percentile of the score, the lowest quartile, as the cutoff point, which is consistent with the methods used in the published literature [39]. Furthermore, considering that age has a significant effect on cognitive performance, the score was further categorized based on age (60 to <70 years, 70 to <80 years, and ≥80 years) [40]. Regarding the CERAD test, the cutoff values of low cognitive performance were 22, 19 and 16 for the three age groups, respectively. Regarding the Animal Fluency test, the cutoff values were 14, 13 and 12, and for the DSST, the cutoff values were 38, 34 and 29, respectively. For each dimension, participants were divided into two groups: the low cognitive performance group, with people who scored lower than the corresponding cutoff values, and the rest, who were assigned to the normal cognitive performance group.

### 2.3. Dietary Intake Assessment

Consumption data for coffee and caffeine intake from coffee came from two cycles of the nationally representative NHANES, corresponding to the years 2011–2012 and 2013–2014. Coffee and caffeine intake from coffee were obtained from two 24-hour dietary recall interviews. Trained interviewers conducted dietary recall interviews using an automated data collection system during the MEC examination [41]. At the end of the MEC dietary interview, the interviewers will schedule the sample persons for a phone follow-up (PFU) interview 3–10 days later. Dietary telephone interviewers at the home office will conduct the PFU interviews. 

We used the United States Department of Agriculture (USDA) Dietary Sources of Nutrients database to identify coffee beverages and caffeine [42]. Coffee consumption was determined using a food frequency questionnaire during the home interview [43,44]. Participants were asked the question “Did you drink coffee?”. If the participants answer affirmatively, they were asked to report the number of cups of coffee they drank by choosing from 10 categories (none, less than 1 cup per month, 1–3 cups per month, 1 cup per week, 2–4 cups per week, 5–6 cups per week, 1 cup per day, 2–3 cups per day, 4–5 cups per day, 6 or more cups per day). They also reported questions about types of coffee beverage, for example, “How often was the coffee you drank decaffeinated?”, “How often did you add sugar or honey to your coffee?”, “What kind of milk was usually added to your coffee?”. The resulting data files provided frequency information for all these questions which can be collapsed to examine intake for all coffee combined, or by type. The classification of coffee beverage is shown in Supplementary Table S1. The agreement between the two recalls was high (intraclass correlation coefficient: 0.634 for total coffee intake, 0.631 for caffeinated coffee intake, 0.612 for decaffeinated coffee intake, 0.676 for caffeine intake), so we used a combination of the first-day and second-day mean values to make use of all available dietary data. Caffeine consumption (mg/day) was categorized into quartiles (Q1: <25th percentile, Q2: ≥25th to 50th percentile, Q3: ≥50th to 75th percentile, Q4: ≥75th percentile) with Q1 as the reference category. Total coffee consumption (g/day) was divided into four groups, but quartiles could not be used to divide coffee consumption because 28% of the subjects reported no coffee intake. Beyond those reporting no coffee intake, coffee drinkers were divided into tertiles, resulting in four categories: (1) no coffee intake, (2) 1–266.4 g/day, (3) 266.4–495 g/day and (4) 495 or more g/day. Decaffeinated coffee consumption (g/day) was divided into two groups: (1) no decaffeinated coffee intake, and (2) more than 0 g/day. Caffeinated coffee consumption (g/day) was divided into three groups: (1) no caffeinated coffee intake, (2) 1–384.8 g/day and (3) 384.8 or more g/day. 

### 2.4. Covariates

In addition to coffee and caffeine intake from coffee, we investigated some potential confounding factors, which included: age (60–70 years, 70–80 years, and ≥80 years), gender (male and female), race (Mexican American, other Hispanic, Non-Hispanic White, Non-Hispanic Black and other races), educational level (below high school, high school and above high school), marital status (married/living with partner and widowed/divorced/separated/never married), poverty–income ratio (≤0.99 and ≥1), body mass index (BMI) (normal: <25 kg/m^2^, overweight: 25 to <30 kg/m^2^, obese: ≥30 kg/m^2^), drinking (having at least 12 alcohol drinks per year or not) and smoking status (never: never smoked or smoked <100 cigarettes in life, former: smoked ≥100 cigarettes in life and currently no longer smoking and current: smoked ≥100 cigarettes in life and currently smoking) [45,46]. Total energy intake was obtained from the 24-hour dietary recall. Poverty–income ratio (PIR)—the ratio of family income to the poverty threshold—was used to define income [47]. History of hypertension, diabetes or stroke was defined as self-reported physician diagnosis of hypertension, diabetes or stroke. 

### 2.5. Statistical Analysis

All statistical analyses were conducted by Stata 15.0 (Stata Corporation, College Station, TX). When combining two 2-year cycles of the continuous data, a new sample weight (the original 2-year sample weight divided by 2) was constructed according to the analytical guidelines of the NHANES [48]. The Kolmogorov–Smirnov normality test was adopted to test the normality of continuous variables, and we described normally distributed variables with mean ± standard deviation (SD) and non-normally distributed variables with median (interquartile range). Student’s t-test was used to compare the mean levels between the low cognitive performance group and the normal cognitive performance group if the variable was normally distributed. The Mann–Whitney U test was adopted if the variable was not normally distributed. Chi-square tests were chosen to compare the percentages of categorical variables between the different groups. 

For the current study, coffee and caffeine consumption were categorized into four groups, caffeinated coffee consumption was categorized into three groups and decaffeinated coffee consumption was categorized into two groups. When treated as a binary variable, cognitive performance was divided into two groups. We conducted binary logistic regression analyses to examine the association of total coffee consumption, caffeinated coffee consumption, decaffeinated coffee consumption and caffeine intake from coffee with cognitive performance. Based on prior studies and theoretical considerations [40,49,50], we selected established risk factors for cognitive performance, which were also known to be associated with coffee intake. Model 1 did not adjust any confounders. Model 2 adjusted for age and gender. Model 3 additionally adjusted for race, educational level, marital status, poverty–income ratio, body mass index, total energy, drinking status, smoking status, diabetes, hypertension and stroke. Additionally, we performed sensitivity analysis by excluding caffeinated coffee consumers in the decaffeinated coffee analysis, and excluding decaffeinated coffee consumers in the caffeinated coffee analysis and caffeine from coffee analysis. We then performed a stratified analysis by gender to examine the associations between caffeine intake from coffee and different dimensions of low cognitive performance, and tested gender as an interaction with caffeine intake in the model that adjusted for the same covariates. We also conducted linear regression analyses to examine the association of total coffee consumption, caffeinated coffee consumption, decaffeinated coffee consumption and caffeine intake from coffee with cognitive performance, leaving independent and dependent variables as continuous variables. We further used restricted cubic spline with three knots located at the 5th, 50th and 95th percentiles of the exposure distribution to assess the dose–response relationship in the logistic regression Model 3. A two-sided *p* < 0.05 was considered statistically significant.

## 3. Results

Of all the participants, there were significant differences (*p* < 0.01) between individuals with low cognitive performance and normal cognitive performance in the distribution of race, educational level, poverty-income ratio, smoking status, diabetes, stroke, total energy intake, coffee intake, caffeinated coffee intake and caffeine intake from coffee among the CERAD test, Animal Fluency test and DSST (Table 1). As can be seen in the tables, those who reported low cognitive performance were more likely to be non-black, current smokers, have lower educational level, poverty-income ratio, less coffee intake, less caffeinated coffee intake, less caffeine intake and higher prevalence of diabetes and stroke than those who reported normal cognitive performance. Participants in the low cognitive performance group who took the CERAD and Animal Fluency test were more likely to be older. For the DSST and Animal Fluency test, the prevalence of hypertension in people with low cognitive performance was significantly higher than that of people with normal cognitive performance, and the alcohol drinking rate was lower in people with low cognitive performance than people with normal cognitive performance. People in the low cognitive performance group with the CERAD and DSST tests tended to be male, whereas the normal cognitive performance people were more likely to be female. 

Table 2 shows the associations between total coffee intake and different dimensions of cognitive performance. Compared to those reporting no coffee consumption, those who reported 266.4–495 (g/day) had a crude odd ratio (OR) with 95% confidence interval (CI) of 0.74(0.50–0.91) for DSST score. After adjustment for age and gender, total coffee intake was still associated with cognitive performance. In Model 3, compared to those reporting no coffee consumption, those who reported 266.4–495 (g/day) had a multivariate-adjusted OR (95% CI) of 0.56(0.35–0.89). 

Table 3 presents the associations of caffeinated coffee and decaffeinated coffee with cognitive performance. Compared to those reporting no caffeinated coffee consumption, those who reported ≥384.8 (g/day) had a crude OR with 95% CI of 0.58(0.42–0.81) for DSST score. After adjustment for age and gender, caffeinated coffee intake was still associated with cognitive performance. In Model 3, compared to those reporting no caffeinated coffee consumption, those who reported ≥384.8 (g/day) had a multivariate-adjusted OR (95% CI) of 0.68(0.48–0.97). No significant association was observed between decaffeinated coffee and different dimensions of cognitive performance. In sensitivity analysis, the association of caffeinated coffee with DSST score was still significant, and the association of decaffeinated coffee with cognitive performance was not significant (Supplementary Table S2).

Table 4 shows the associations between caffeine intake from coffee and different dimensions of cognitive performance. Compared to the lowest quartile of caffeine intake from coffee, the crude OR (95% CI) of the quartile (Q) three was 0.60(0.40–0.91) for CERAD test score. After adjustment for age and gender, caffeine intake was still associated with cognitive performance. In Model 3, compared to the lowest quartile of caffeine intake from coffee, the multivariate adjusted OR (95% CI) of the quartile (Q) three was 0.62(0.38–0.98). In sensitivity analysis, in the fully adjusted model, the negative associations of caffeine from coffee with CERAD test score and DSST score were significant (Supplementary Table S3). 

We also evaluated the associations between caffeine intake from coffee and cognitive performance among men and women, separately, to assess potential differences by gender (Supplementary Table S4). For women, caffeine intake was associated with CERAD test score and DSST score, the corresponding ORs (95% CIs) were 0.34(0.17–0.65) and 0.39(0.20–0.76) in Model 3. For men, there was no significant association between caffeine intake and different dimensions of cognitive performance in Model 3. A statistically significant interaction was noted between caffeine from coffee and gender in the CERAD test in the model that adjusted for the same covariates (*p* = 0.036).

The results of linear regression analyses of associations between total coffee consumption, caffeinated coffee consumption, decaffeinated coffee consumption, caffeine from coffee and cognitive performance is shown in the Supplementary Materials (Tables S5–S7). In the fully adjusted model, there was significant association between coffee consumption and DSST score (β = 0.0017, 95% CI: 0.0001–0.003). Moreover, the associations of caffeinated coffee with Animal Fluency test score (β = 0.0006, 95% CI: 0.00001–0.0013) and DSST score (β = 0.0021, 95% CI: 0.0003–0.004) were significant in Model 3. No significant association was observed between decaffeinated coffee and different dimensions of cognitive performance. Furthermore, there was a significant association between caffeine from coffee and CERAD test score (β = 0.0025, 95% CI: 0.0001–0.0049) in Model 3.

**Figure 2:**
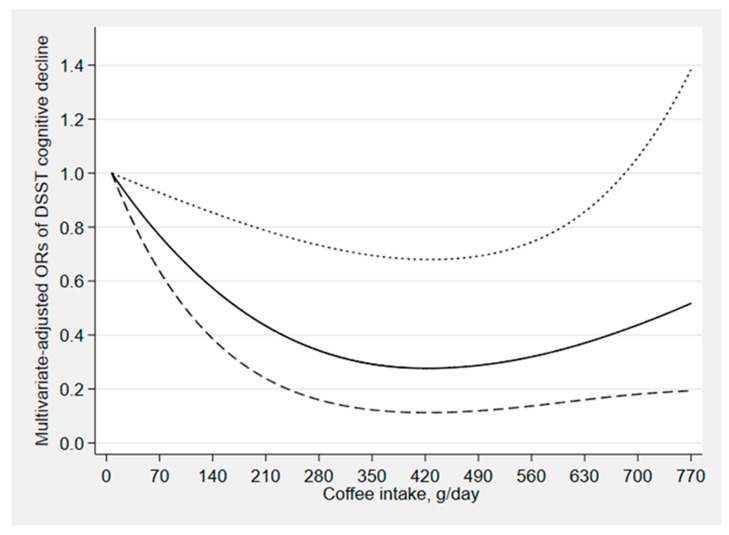
Dose–response relationship between coffee intake and DSST score. The solid line and dashed line represent the estimated ORs and their 95% confidence intervals (OR, odds ratio).

**Figure 3:**
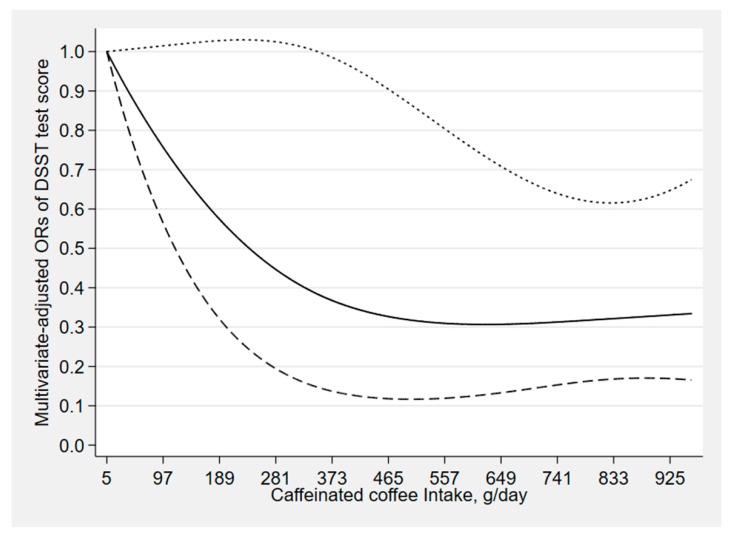
Dose–response relationship between caffeinated coffee and DSST score. The solid line and dashed line represent the estimated ORs and their 95% confidence intervals (OR, odds ratio).

**Figure 4:**
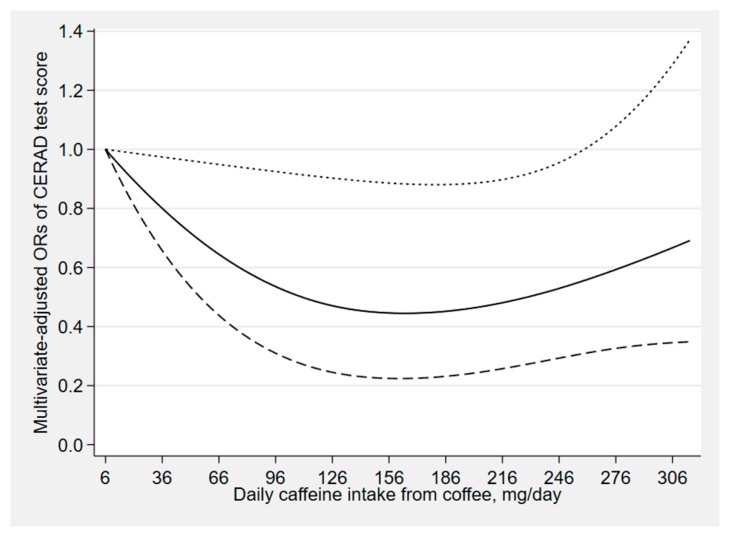
Dose–response relationship between caffeine intake from coffee and CERAD test score. The solid line and dashed line represent the estimated ORs and their 95% confidence intervals (OR, odds ratio).

Figure 2, Figure 3 and Figure 4 depict the results of the restricted cubic spline analyses. We found a suggestion of L-shaped associations of total coffee intake and caffeinated coffee intake with DSST score. The prevalence of low cognitive performance decreased with increasing intake of total coffee and caffeinated coffee and showed a nonlinear dose–response relationship (*p*_total coffee_ for nonlinearity = 0.039, *p*_caffeinated coffee_ for nonlinearity = 0.023). We also found a suggestion of L-shaped associations between caffeine intake from coffee and CERAD test score. The prevalence of low cognitive performance decreased with increasing intake of caffeine and showed a nonlinear dose–response relationship (*p*_caffeine_ for nonlinearity = 0.032).

## 4. Discussion

In this study, we combined data from NHANES 2011–2012 and 2013–2014 and included 2513 Americans aged 60 years or older. In the fully adjusted model, the associations of total coffee, caffeinated coffee and caffeine intake from coffee with DSST score and CERAD test score were significant, and L-shaped dose–response relationships were also detected. No significant association was observed between decaffeinated coffee and different dimensions of cognitive performance. In sensitivity analyses, the associations of caffeinated coffee and caffeine from coffee with DSST score and CERAD test score were still significant by excluding decaffeinated coffee consumers. The association of decaffeinated coffee with cognitive performance was not significant by excluding caffeinated coffee consumers. In stratified analyses, higher levels of caffeine intake from coffee were associated with higher CERAD test score and DSST score in women but not in men.

Our finding about coffee consumption was partially consistent with the findings from some previous studies [11,12,13,20,51]. A population-based study of 145 community-based older individuals [11] found a positive effect of coffee on cognitive performance. In addition, a recent 30-year follow-up study of 8000 Japanese-American men [12] suggested that coffee intake might protect against Parkinson’s disease. Alzheimer’s and Parkinson’s diseases are both neurodegenerative, approximately thirty percent of Parkinson’s disease patients might develop an Alzheimer’s-like dementia and thirty percent of Alzheimer’s patients might develop Parkinson’s-like changes [52]. In a large, population-based study of 9003 British people, Jarvis [53] found a significant positive trend between coffee intake and cognitive performance. Furthermore, studies of the association between coffee and cognitive performance also indicated that although reduced risk was related to coffee consumption in men [51], the effect was more pronounced in women [19,20], whereas some studies [54,55,56,57] showed null or adverse associations. A population-based Rotterdam study [55] of 2914 participants in a five-year follow-up, and a cohort study of 14,563 participants (35–74 years old) conducted by Araújo [56], showed null or adverse effects of coffee consumption on cognitive performance. 

We also found that caffeinated coffee and caffeine from coffee were associated with cognitive performance, which were consistent with previous studies. A population-based cohort study of 7017 community-based older individuals [19] showed that caffeine intake from coffee appeared to reduce cognitive decline. In a placebo-controlled cross-over design [27], caffeine intake from coffee was also found to have a protective effect on cognitive performance. Moreover, a meta-analysis of eleven observational studies [58] also suggested a positive effect of caffeine from coffee on cognitive performance, with a summary relative risk (RR) of 0.84 (95% CI: 0.72–0.99, I2 = 42.6%). However, a meta-analysis of observational studies found that caffeine intake from coffee was not associated with the risk of cognitive disorders [28].

In addition, no significant association between decaffeinated coffee and cognitive performance was found in our study, likely reflecting lower statistical power for these analyses or due to the small number of participants. The finding was partially consistent with those of the population-based study of 1528 elderly people, conducted by Johnson-Kozlow [25], which found a positive effect of caffeinated coffee on cognitive performance, and there was no significant association between decaffeinated coffee intake and cognitive function. Moreover, a placebo-controlled trial of sixty older individuals suggested that no significant association was observed between decaffeinated coffee and cognitive function [26]. In contrast, a randomized placebo-controlled study indicated that decaffeinated coffee might have a protective effect on cognitive performance [23]. An animal trial conducted by Jang et al. also provided evidence that decaffeinated coffee might prevent memory impairment in humans [24].

The mechanisms of the relationship between caffeine intake and cognitive performance remained unclear, but there have been several possibilities. Caffeine may have the ability to induce mRNA and protein expression and mediate NF-E2-related factor 2-Antioxidant Response Element (Nrf2-ARE) pathway stimulation, which could improve the overall antioxidant capacity of the body and thus contribute to ameliorating oxidative stress, inflammation and carcinogenesis [1]. Moreover, Riedel et al. [27] reversed the effects of scopolamine through the administration of 250 mg of caffeine and concluded that caffeine acted through cholinergic pathways and specifically enhanced memory. Furthermore, caffeine and its metabolites helped in proper cognitive performance. Coffee lipid fraction containing cafestol and kahweol played a protective role against some malignant cells by regulating the detoxifying enzymes. 

The differences found between men and women indicated that women were more vulnerable to the effects of caffeine than men. The elimination half-life of caffeine ranged from three to seven hours; however, elimination was about twenty percent shorter because of more rapid biotransformation among women [59]. Research by Carrillo [60] indicated that women were more likely than men to experience acute toxic reactions, such as restlessness, palpitation, muscle tremor and dizziness, after taking high doses of caffeine. Therefore, gender differences may be due to pharmacodynamic differences in sensitivity of men and women to caffeine effects. In another study, Relling et al. [61] indicated that healthy women had higher levels of xanthine oxidase activity than did men after ingesting equal amounts of caffeine, suggesting that men and women metabolized caffeine differently. A randomized controlled trial also provided evidence of different responses of men and women to caffeine, which may be mediated by changes in circulating steroid hormones [62].

Our study presents several advantages. A major strength was the use of a large nationally representative sample of older adults in the United States. In terms of survey methods and quality control, the NHANES was high quality. In addition, wide ranges of potential confounders were controlled to provide a better estimate of the association of coffee and caffeine intake with cognitive performance. Moreover, we investigated the dose–response relationship of coffee and caffeine consumption with cognitive performance. 

We acknowledge several limitations of our study. Primarily, as a cross-sectional study, these associations cannot necessarily be considered as causality, so it was difficult to generalize the results of this study to the causal relationship from coffee consumption to cognitive performance. Furthermore, the cognitive tests, chosen for ease of administration, availability and use in other surveys, did not cover all domains of cognitive function. Adults who performed well in one domain may not perform well in another domain. What is more, we cannot rule out the co-linearity effect in this study. Finally, the dietary data was obtained from two 24-hour dietary recall interviews, which did not accurately reflect individuals’ usual intake, but some studies have shown that two 24-h recalls might be sufficient to assess the daily dietary intake [63].

## 5. Conclusions

Our study suggests that coffee, caffeinated coffee and caffeine intake from coffee were associated with cognitive performance for participants aged 60 years or older in the United States. However, no significant association was observed between decaffeinated coffee and cognitive performance. Large-scale, prospective studies are needed to further elucidate the effects of decaffeinated coffee on cognitive performance among older adults.

## Figures and Tables

**Table 1:** Characteristics of the study population, National Health and Nutrition Examination Survey (NHANES) 2011–2014 (N = 2513)

		CERAD Test			Animal Fluency Test			Digit Symbol Test		
	Number of Subjects (N)	Normal Cognitive Performance	Low Cognitive Performance	*p* Value	Normal Cognitive Performance	Low Cognitive Performance	*p* Value	Normal Cognitive Performance	Low Cognitive Performance	*p* Value
**Number of subjects (%) ^1^**		1800(71.6)	713(28.4)		1789(71.2)	724(28.8)		1857(73.9)	656(26.1)	
**Age (%) ^1^**	2513			0.336			0.016			0.920
**60–70 years**		959(53.3)	403(56.5)		961(53.7)	401(55.4)		1009(54.3)	353(53.8)	
**70–80 years**		546(30.3)	200(28.1)		557(31.1)	189(26.1)		552(29.7)	194(29.6)	
**≥80 years**		295(16.4)	110(15.4)		271(15.1)	134(18.5)		296(15.9)	109(16.6)	
**Gender (%) ^1^**	2513			<0.01			0.741			<0.01
**Male**		782(43.4)	432(60.6)		868(48.5)	346(47.8)		843(45.4)	371(56.6)	
**Female**		1018(56.6)	281(39.4)		921(51.5)	378(52.2)		1014(54.6)	285(43.4)	
**Race (%) ^1^**	2513			<0.01			<0.01			<0.01
**Mexican American**		129(7.2)	82(11.5)		149(8.3)	62(8.6)		120(6.5)	91(13.9)	
**Other Hispanic**		143(7.9)	101(14.2)		151(8.4)	93(12.8)		120(6.5)	124(18.9)	
**Non-Hispanic White**		985(54.7)	279(39.1)		1031(57.6)	233(32.2)		1093(58.9)	171(26.1)	
**Non-Hispanic Black**		384(21.3)	204(28.6)		333(18.6)	255(35.2)		342(18.4)	246(37.5)	
**Other race**		159(8.8)	47(6.6)		125(7.0)	81(11.2)		182(9.8)	24(3.7)	
**Educational level (%) ^1^**	2511			<0.01			<0.01			<0.01
**Below high school**		320(17.8)	277(38.9)		329(18.4)	268(37.1)		244(13.1)	353(54.0)	
**High school**		423(23.5)	174(24.4)		405(22.6)	192(26.6)		441(23.7)	156(23.9)	
**Above high school**		1056(58.7)	261(36.7)		1055(59.0)	262(36.3)		1172(63.1)	145(22.2)	
**Material status (%) ^1^**	2510			0.889			0.032			<0.01
**Married/living with partner**		1056(58.7)	416(58.4)		1072(60.0)	400(55.3)		1144(61.7)	328(50.1)	
**Widowed/divorced/separated/never married**		742(41.3)	296(41.6)		715(40.0)	323(44.7)		711(38.3)	327(49.9)	
**Poverty–income ratio (%) ^1^**	2324			<0.01			<0.01			<0.01
**≤0.99**		224(13.4)	144(22.0)		217(13.0)	151(22.9)		185(10.7)	183(30.6)	
**≥1**		1445(86.6)	511(78.0)		1449(87.0)	507(77.1)		1540(89.3)	416(69.4)	
**Body mass index (%) ^1^**	2483			0.055			0.638			0.498
**< 25 kg/m^2^**		460(25.9)	200(28.3)		464(26.1)	196 (27.7)		487(26.4)	173(27.2)	
**25-30 kg/m^2^**		603(33.9)	259(36.7)		625(35.2)	237(33.5)		653(35.4)	209(32.8)	
**≥30 kg/m^2^**		714(40.2)	247(35.0)		686(38.6)	275(38.8)		706(38.2)	255(40.0)	
**Smoking status (%) ^1^**	2510			0.003			0.018			<0.01
**Never**		904(50.3)	339(47.5)		874(48.9)	369(51.0)		927(49.9)	316(48.2)	
**Former**		708(39.4)	265(37.2)		719(40.2)	254(35.0)		743(40.0)	230(35.1)	
**Current**		187(10.3)	109(15.3)		195(10.9)	101(14.0)		186(10.1)	110(16.7)	
**Hypertension (%) ^1^**	2510	1123(62.4)	445(62.6)	0.939	1077(60.3)	491(67.9)	<0.01	1119(60.4)	449(68.4)	<0.01
**Diabetes (%) ^1^**	2511	392(21.8)	197(27.6)	0.002	377(21.1)	212(29.3)	<0.01	373(20.1)	216(33.0)	<0.01
**Had at least 12 alcohol drinks/year (%) ^1^**	2495	1237(69.0)	491(69.9)	0.643	1270(71.4)	458(64.0)	<0.01	1322(71.5)	406(62.8)	<0.01
**Ever told you had a stroke (%) ^1^**	2508	107(6.0)	59(8.3)	0.036	100(5.6)	66(9.1)	0.001	91(4.9)	75(11.4)	<0.01
**Coffee intake (g/day) ^2^**	2513	255.77(499.25)	228.9(442.5)	0.017	273.8(510)	212.8(397.5)	<0.01	277.5(510)	208.43(393.2)	<0.01
**Caffeinated coffee intake (g/day) ^2^**	2513	240(472.5)	211(429.1)	0.025	247.5(480)	195.5(363.5)	<0.01	251.6(480)	192.4(370.975)	<0.01
**Decaffeinated coffee intake (g/day) ^2^**	2513	74.1(221)	63(180.6)	0.919	71.1(207)	70.4(218)	0.327	71.7(208)	68.7(219)	0.412
**Caffeine intake from coffee (mg/day) ^2^**	1803	131.75 (213.03)	107 (135)	0.003	140 (149.5)	99 (129.75)	<0.01	140 (147.5)	95 (126.875)	<0.01
**Total energy intake (kcal/day) ^2^**	2513	1754(816.25)	1647.5(909.5)	<0.01	1796(838)	1583.5(837)	<0.01	1796(815.5)	1560.25(894)	<0.01

Data is number of subjects (percentage) or medians (interquartile ranges); ^1^ Chi-square test was used to compare the percentage between participants with and without low cognitive performance; ^2^ Mann-Whitney U test was used to compare the median values between participants with and without low cognitive performance.

**Table 2:** Weighted odds ratios (95% confidence intervals) for scores on the Consortium to Establish a Registry for Alzheimer’s Disease (CERAD) test, Animal Fluency test and Digit Symbol Substitution Test (DSST) across quartiles of coffee intake, NHANES 2011–2014 (N = 2513).

	CERAD Test				Animal Fluency Test				DSST			
Coffee (g/day)	Case/Participants	Model 1 ^1^	Model 2 ^1^	Model 3 ^1^	Case/Participants	Model 1 ^1^	Model 2 ^1^	Model 3 ^1^	Case/Participants	Model 1 ^1^	Model 2 ^1^	Model 3 ^1^
0	210/710	1.00 (Ref.)	1.00 (Ref.)	1.00 (Ref.)	219/710	1.00 (Ref.)	1.00 (Ref.)	1.00 (Ref.)	198/710	1.00 (Ref.)	1.00 (Ref.)	1.00 (Ref.)
1 to <266.4	196/602	1.23(0.78–1.97)	1.32(0.83–2.08)	1.24(0.72–2.14)	212/602	1.16(0.79–1.69)	1.11(0.77–1.61)	0.98(0.66–1.44)	205/602	1.39(0.98–1.97)	1.36(0.96–1.93)	1.19(0.74–1.90)
266.4 to <495	165/607	0.68(0.44–1.06)	0.68(0.42–1.08)	0.71(0.44–1.13)	166/607	0.70(0.48–1.03)	0.66(0.46–0.95)	0.85(0.61–1.18)	148/607	0.74(0.50–0.91) *	0.71(0.47–0.87) *	0.56(0.35–0.89) *
≥495	142/594	0.80(0.50–1.27)	0.75(0.45–1.23)	0.89(0.52–1.54)	127/594	0.71(0.48–1.07)	0.72(0.48–1.06)	1.13(0.73–1.76)	105/594	0.57(0.40–0.81) *	0.56(0.39–0.79) *	1.03(0.67–1.62)

^1^ Calculated using binary logistic regression; Reference (Ref.); Model 2 adjusted for age and gender; Model 3 adjusted for age and gender, race, educational level, marital status, income, body mass index (BMI), energy, drinking status, smoking status, hypertension, diabetes, and stroke. * *p* < 0.05.

**Table 3:** Weighted odds ratios (95% confidence intervals) for scores on the Consortium to Establish a Registry for Alzheimer’s Disease (CERAD) test, Animal Fluency test and Digit Symbol Substitution Test (DSST) across caffeinated coffee and decaffeinated coffee, NHANES 2011–2014 (N = 2513).

	CERAD Test				Animal Fluency Test				DSST			
	Case/Participants	Model 1 ^1^	Model 2 ^1^	Model 3 ^1^	Case/Participants	Model 1 ^1^	Model 2 ^1^	Model 3 ^1^	Case/Participants	Model 1 ^1^	Model 2 ^1^	Model 3 ^1^
Caffeinated coffee (g/day)												
0	291/958	1.00 (Ref.)	1.00 (Ref.)	1.00 (Ref.)	297/958	1.00 (Ref.)	1.00 (Ref.)	1.00 (Ref.)	276/958	1.00 (Ref.)	1.00 (Ref.)	1.00 (Ref.)
1 to <384.8	226/784	0.91(0.65–1.28)	0.93(0.66–1.32)	0.94(0.65–1.35)	256/784	0.96(0.71–1.32)	0.94(0.70–1.27)	0.99(0.71–1.40)	224/784	1.03(0.74–1.44)	1.01(0.71–1.45)	1.04(0.71–1.52)
≥384.8	196/771	0.78(0.50–1.21)	0.72(0.45–1.16)	0.82(0.51–1.30)	171/771	0.76(0.50–1.14)	0.77(0.51–1.16)	0.92(0.61–1.39)	156/771	0.58(0.42–0.81) **	0.56(0.41–0.80) **	0.68(0.48–0.97) *
Decaffeinated coffee (g/day)												
0	591/2094	1.00 (Ref.)	1.00 (Ref.)	1.00 (Ref.)	593/2094	1.00 (Ref.)	1.00 (Ref.)	1.00 (Ref.)	537/2094	1.00 (Ref.)	1.00 (Ref.)	1.00 (Ref.)
>0	122/419	0.92(0.43–1.96)	1.02(0.45–2.33)	1.45(0.75–2.80)	131/419	0.99(0.50–1.95)	0.92(0.45–1.87)	1.09(0.54–2.13)	119/419	1.32(0.63–2.77)	1.31(0.60–2.86)	1.84(0.68–3.17)

^1^ Calculated using binary logistic regression; Reference (Ref.); Model 2 adjusted for age and gender; Model 3 adjusted for age and gender, race, educational level, marital status, income, body mass index (BMI), energy, drinking status, smoking status, hypertension, diabetes, and stroke. * *p* < 0.05; ** *p* < 0.01.

**Table 4:** Weighted odds ratios (95% confidence intervals) for scores on the Consortium to Establish a Registry for Alzheimer’s Disease (CERAD) test, Animal Fluency test and Digit Symbol Substitution Test (DSST) across quartiles of caffeine intake from coffee, NHANES 2011–2014 (N = 1803).

	CERAD Test				Animal Fluency Test				DSST			
	Case/Participants	Model 1 ^1^	Model 2 ^1^	Model 3 ^1^	Case/Participants	Model 1 ^1^	Model 2 ^1^	Model 3 ^1^	Case/Participants	Model 1 ^1^	Model 2 ^1^	Model 3 ^1^
Caffeine (mg/day)												
<67	150/455	1.00 (Ref.)	1.00 (Ref.)	1.00 (Ref.)	162/455	1.00 (Ref.)	1.00 (Ref.)	1.00 (Ref.)	160/455	1.00 (Ref.)	1.00 (Ref.)	1.00 (Ref.)
67 to <124.5	133/450	0.77 (0.50–1.20)	0.74(0.49–1.13)	0.80(0.48–1.33)	146/450	1.22(0.75–1.96)	1.24(0.78–1.99)	1.36(0.80–2.34)	123/450	0.75(0.46–1.20)	0.74(0.46–1.17)	0.78(0.45–1.33)
124.5 to <208	112/448	0.60(0.40–0.91) *	0.53(0.34–0.83) **	0.62(0.38–0.98) *	108/448	0.66(0.44–0.97) *	0.65(0.45–0.95) *	0.88(0.59–1.33)	98/448	0.59(0.31–1.13)	0.55(0.31–0.98) *	0.67(0.39–1.15)
≥208	108/450	0.76(0.52–1.11)	0.67(0.45–0.99) *	0.92(0.62–1.36)	89/450	0.76(0.43–1.34)	0.83(0.49–1.40)	1.28(0.74–2.24)	77/450	0.42(0.25–0.71) **	0.39(0.23–0.67) **	0.83(0.48–1.47)

^1^ Calculated using binary logistic regression; Reference (Ref.); Model 2 adjusted for age and gender; Model 3 adjusted for age and gender, race, educational level, marital status, income, body mass index (BMI), energy, drinking status, smoking status, hypertension, diabetes, and stroke. * *p* < 0.05; ** *p* < 0.01.

## Supplementary Materials

The following are available online at https://www.mdpi.com/2072-6643/12/3/840/s1, **Table S1**: Coffee type and caffeinated status for each coffee item identified by USDA food code in dietary recall. **Table S2**: Weighted odds ratios (95% confidence intervals) for scores on CERAD test, Animal Fluency test and DSST across caffeinated coffee (N = 2094) and decaffeinated coffee (N = 958) in sensitivity analysis. **Table S3**: Weighted odds ratios (95% confidence intervals) for scores on CERAD test, Animal Fluency test and DSST across quartiles of caffeine intake from coffee, excluding decaffeinated coffee consumers (N = 1384). **Table S4**: Weighted odds ratios (95% confidence intervals) for scores on CERAD test, Animal fluency test and DSST across quartiles of caffeine intake from coffee, stratified by gender (N =1803). **Table S5**: Weighted Regression coefficients (β) and 95% confidence intervals for scores on CERAD test, Animal Fluency test and DSST across total coffee intake (N = 2513). **Table S6**: Weighted Regression coefficients (β) and 95% confidence intervals for scores on CERAD test, Animal Fluency test and DSST across caffeinated coffee and decaffeinated coffee (N = 2513). **Table S7**: Weighted Regression coefficients (β) and 95% confidence intervals for scores on CERAD test, Animal Fluency test and DSST across caffeine intake from coffee (N = 1803).
