# Supplementary material for: Association of Coffee, Decaffeinated Coffee and Caffeine Intake from Coffee with Cognitive Performance in Older Adults: National Health and Nutrition Examination Survey (NHANES) 2011–2014
Source: Nutrients. 2020 Mar 20;12(3):840. doi: 10.3390/nu12030840 (PMC7146118; doi:10.3390/nu12030840)
Supplement: Supplementary file 1 [file nutrients-12-00840-s001.pdf]

Table 1. Coffee type and caffeinated status for each coffee item identified by USDA food code in dietary recall, NHANES 2011-2014.

USDA food code	USDA food descriptions	Caffeinated status [a]
92100000	Coffee, NS as to type	C
92100500	Coffee, NS as to brewed or instant	C
92101000	Coffee, brewed	C
92101500	Coffee, brewed, blend of regular and decaffeinated	D
92101600	Coffee, Turkish	C
92101610	Coffee, espresso	C
92101630	Coffee, espresso, decaffeinated	D
92101700	Coffee, brewed, flavored	C
92101800	Coffee, Cuban	C
92101810	Coffee, macchiato	C
92101820	Coffee, macchiato, sweetened	C
92101850	Coffee, cafe con leche	C
92101851	Coffee, cafe con leche, decaffeinated	D
92101900	Coffee, Latte	C
92101901	Coffee, Latte, nonfat	C
92101903	Coffee, Latte, with non-dairy milk	C
92101904	Coffee, Latte, flavored	C
92101905	Coffee, Latte, nonfat, flavored	C
92101906	Coffee, Latte, with non-dairy milk, flavored	C
92101910	Coffee, Latte, decaffeinated	D
92101911	Coffee, Latte, decaffeinated, nonfat	D
92101913	Coffee, Latte, decaffeinated, with non-dairy milk	D
92101917	Coffee, Latte, decaffeinated, flavored	D
92101918	Coffee, Latte, decaffeinated, nonfat, flavored	D

92101919	Coffee, Latte, decaffeinated, with non-dairy milk, flavored	D
92101920	Frozen coffee drink	C
92101921	Frozen coffee drink, nonfat	C
92101923	Frozen coffee drink, with non-dairy milk	C
92101925	Frozen coffee drink, with whipped cream	C
92101926	Frozen coffee drink, nonfat, with whipped cream	C
92101928	Frozen coffee drink, with non-dairy milk and whipped cream	C
92101930	Frozen coffee drink, decaffeinated	D
92101931	Frozen coffee drink, decaffeinated, nonfat	D
92101933	Frozen coffee drink, decaffeinated, with non-dairy milk	D
92101935	Frozen coffee drink, decaffeinated, with whipped cream	D
92101936	Frozen coffee drink, decaffeinated, nonfat, with whipped cream	D
92101938	Frozen coffee drink, decaffeinated, with non-dairy milk and whipped cream	D
92101950	Coffee, Cafe Mocha	C
92101955	Coffee, Cafe Mocha, nonfat	C
92101960	Coffee, Cafe Mocha, with non-dairy milk	C
92101965	Coffee, Cafe Mocha, decaffeinated	D
92101970	Coffee, Cafe Mocha, decaffeinated, nonfat	D
92101975	Coffee, Cafe Mocha, decaffeinated, with non-dairy milk	D
92102000	Frozen mocha coffee drink	C
92102010	Frozen mocha coffee drink, nonfat	C
92102020	Frozen mocha coffee drink, with non-dairy milk	C

92102030	Frozen mocha coffee drink, with whipped cream	C
92102040	Frozen mocha coffee drink, nonfat, with whipped cream	C
92102050	Frozen mocha coffee drink, with non-dairy milk and whipped cream	C
92102060	Frozen mocha coffee drink, decaffeinated	D
92102070	Frozen mocha coffee drink, decaffeinated, nonfat	D
92102080	Frozen mocha coffee drink, decaffeinated, with non-dairy milk	D
92102090	Frozen mocha coffee drink, decaffeinated, with whipped cream	D
92102100	Frozen mocha coffee drink, decaffeinated, nonfat, with whipped cream	D
92102110	Frozen mocha coffee drink, decaffeinated, with non-dairy milk and whipped cream	D
92102400	Iced Coffee, brewed	C
92102401	Iced Coffee, brewed, decaffeinated	D
92102450	Iced Coffee, pre-lightened and pre-sweetened	C
92102500	Coffee, Iced Latte	C
92102501	Coffee, Iced Latte, nonfat	C
92102502	Coffee, Iced Latte, with non-dairy milk	C
92102503	Coffee, Iced Latte, flavored	C
92102504	Coffee, Iced Latte, nonfat, flavored	C
92102505	Coffee, Iced Latte, with non-dairy milk, flavored	C
92102510	Coffee, Iced Latte, decaffeinated	D
92102511	Coffee, Iced Latte, decaffeinated, nonfat	D
92102512	Coffee, Iced Latte, decaffeinated, with non-dairy milk	D

92102513	Coffee, Iced Latte, decaffeinated, flavored	D
92102514	Coffee, Iced Latte, decaffeinated, nonfat, flavored	D
92102515	Coffee, Iced Latte, decaffeinated, with non-dairy milk, flavored	D
92102600	Coffee, Iced Cafe Mocha	C
92102601	Coffee, Iced Cafe Mocha, nonfat	C
92102602	Coffee, Iced Cafe Mocha, with non-dairy milk	C
92102610	Coffee, Iced Cafe Mocha, decaffeinated	D
92102611	Coffee, Iced Cafe Mocha, decaffeinated, nonfat	D
92102612	Coffee, Iced Cafe Mocha, decaffeinated, with non-dairy milk	D
92103000	Coffee, instant, reconstituted	C
92104000	Coffee, instant, 50% less caffeine, reconstituted	C
92111000	Coffee, NS as to brewed or instant, decaffeinated	D
92111010	Coffee, brewed, decaffeinated	D
92114000	Coffee, instant, decaffeinated, reconstituted	D
92121000	Coffee, instant, pre-lightened and pre-sweetened with sugar, reconstituted	C
92121001	Coffee, instant, decaffeinated, pre-lightened and pre-sweetened with sugar, reconstituted	D
92121010	Coffee, instant, pre-sweetened with sugar, reconstituted	C
92121020	Coffee, mocha, instant, pre-lightened and pre-sweetened with sugar, reconstituted	C
92121030	Coffee, mocha, instant, pre-lightened and pre-sweetened with low calorie sweetener, reconstituted	C
92121040	Coffee, instant, pre-lightened and pre-sweetened with	C

	low calorie sweetener, reconstituted	
92121041	Coffee, instant, decaffeinated, pre-lightened and pre-sweetened with low calorie sweetener, reconstituted	D
92121050	Coffee, mocha, instant, decaffeinated, pre-lightened and pre-sweetened with low calorie sweetener, reconstituted	D
92130000	Coffee, pre-lightened and pre-sweetened with sugar	C
92130001	Coffee, decaffeinated, pre-lightened and pre-sweetened with sugar	D
92130005	Coffee, pre-lightened and pre-sweetened with low calorie sweetener	C
92130006	Coffee, decaffeinated, pre-lightened and pre-sweetened with low calorie sweetener	D
92130010	Coffee, pre-lightened	C
92130011	Coffee, decaffeinated, pre-lightened	D
92130020	Coffee, pre-sweetened with sugar	D
92130021	Coffee, decaffeinated, pre-sweetened with sugar	C
92130030	Coffee, pre-sweetened with low calorie sweetener	C
92130031	Coffee, decaffeinated, pre-sweetened with low calorie sweetener	D
92152000	Coffee and chicory, brewed	C
92152010	Coffee and chicory, brewed, decaffeinated	D
92161000	Coffee, Cappuccino	C
92161001	Coffee, Cappuccino, nonfat	C
92161002	Coffee, Cappuccino, with non-dairy milk	C
92162000	Coffee, Cappuccino, decaffeinated	D
92162001	Coffee, Cappuccino, decaffeinated, nonfat	D

92162002	Coffee, Cappuccino, decaffeinated, with non-dairy milk	D
92171000	Coffee, bottled/canned	C
92171010	Coffee, bottled/canned, light	C
92191100	Coffee, instant, not reconstituted	C
92191105	Coffee, instant, 50% less caffeine, not reconstituted	C
92191200	Coffee, instant, decaffeinated, not reconstituted	D
92191400	Coffee, instant, pre-sweetened with sugar, not reconstituted	C
92192000	Coffee, mocha, instant, pre-lightened and pre-sweetened with sugar, not reconstituted	C
92192030	Coffee, mocha, instant, pre-lightened and pre-sweetened with low calorie sweetener, not reconstituted	C
92192040	Coffee, mocha, instant, decaffeinated, pre-lightened and pre-sweetened with low calorie sweetener, not reconstituted	D
92193000	Coffee, instant, pre-lightened and pre-sweetened with sugar, not reconstituted	C
92193005	Coffee, instant, decaffeinated, pre-lightened and pre-sweetened with sugar, not reconstituted	D
92193020	Coffee, instant, pre-lightened and pre-sweetened with low calorie sweetener, not reconstituted	C
92193025	Coffee, instant, decaffeinated, pre-lightened and pre-sweetened with low calorie sweetener, not reconstituted	D

[a] Caffeinated status: D, decaffeinated coffee; C, caffeinated coffee

Table 2. Weighted odds ratios (95% confidence intervals) for scores on CERAD test, Animal Fluency test, and DSST test across caffeinated coffee (N = 2094) and decaffeinated coffee (N = 958) in sensitivity analysis, NHANES 2011–2014.

	**CERAD test**			Animal Fluency test			DSST		
	**Model 1[1]**	**Model 2[1]**	Model 3[1]	Model 1[1]	Model 2[1]	Model 3[1]	Model 1[1]	Model 2[1]	Model 3[1]
Caffeinated coffee (g/day)									
0	1.00 (Ref.)	1.00 (Ref.)	1.00 (Ref.)	1.00 (Ref.)	1.00 (Ref.)	1.00 (Ref.)	1.00 (Ref.)	1.00 (Ref.)	1.00 (Ref.)
1 to <384.8	0.95(0.60-1.48)	0.96(0.61-1.53)	0.96 (0.58-1.57)	0.95(0.65-1.37)	0.91(0.64-1.29)	0.96(0.66-1.41)	1.10(0.75-1.60)	1.05(0.70-1.57)	1.09(0.71-1.68)
≥384.8	0.83(0.52-1.34)	0.78(0.48-1.28)	0.89(0.55-1.43)	0.75(0.49-1.14)	0.74(0.48-1.12)	0.89(0.59-1.35)	0.59(0.42-0.85) **	0.57(0.40-0.82) **	0.65(0.45-0.93) *
Decaffeinated coffee (g/day)									
0	1.00 (Ref.)	1.00 (Ref.)	1.00 (Ref.)	1.00 (Ref.)	1.00 (Ref.)	1.00 (Ref.)	1.00 (Ref.)	1.00 (Ref.)	1.00 (Ref.)
>0	0.91(0.41-1.95)	1.01(0.44-2.33)	1.31(0.61-2.74)	0.94(0.48-1.87)	0.87(0.41-1.80)	1.01(0.45-1.97)	1.11(0.51-2.41)	1.09(0.47-2.23)	1.76(0.58-2.87)

Abbreviations: The Consortium to Establish a Registry for Alzheimer's Disease (CERAD); Digit Symbol Substitution Test (DSST).
[1]Calculated using binary logistic regression
Model 2 adjusted for age and gender
Model 3 adjusted for age and gender, race, educational level, marital status, income, BMI, energy, drinking status, smoking status, hypertension, diabetes, and stroke
*p <0.05; **p <0.01

Table S3. Weighted odds ratios (95% confidence intervals) for scores on CERAD test, Animal Fluency test, and DSST test across quartiles of caffeine intake from coffee excluding decaffeinated coffee consumers, NHANES 2011–2014 (N = 1384).

	**CERAD test**			Animal Fluency test			DSST		
	**Model 1[1]**	**Model 2[1]**	Model 3[1]	Model 1[1]	Model 2[1]	Model 3[1]	Model 1[1]	Model 2[1]	Model 3[1]
Caffeine (mg/day)									
<67	1.00 (Ref.)	1.00 (Ref.)	1.00 (Ref.)	1.00 (Ref.)	1.00 (Ref.)	1.00 (Ref.)	1.00 (Ref.)	1.00 (Ref.)	1.00 (Ref.)
67 to <124.5	0.69(0.38-1.25)	0.67(0.38-1.20)	0.70(0.36-1.35)	1.27(0.77-2.07)	1.29(0.78-2.11)	1.48(0.81-2.71)	0.54(0.32-0.91) *	0.52(0.31-0.87) *	0.51(0.23-1.13)
124.5 to <208	0.50(0.28-0.88) *	0.44(0.24-0.83) *	0.49(0.26-0.92) *	0.65(0.35-1.19)	0.62(0.34-1.14)	0.74(0.41-1.32)	0.38(0.18-0.79) *	0.35(0.18-0.67) **	0.30(0.14-0.61) **
≥208	0.63(0.41-0.96) *	0.58(0.37-0.91) *	0.79(0.50-1.23)	0.78(0.38-1.63)	0.83(0.41-1.65)	1.05(0.56-1.97)	0.28(0.15-0.51) **	0.26(0.14-0.49) **	0.35(0.16-0.77) *

Abbreviations: The Consortium to Establish a Registry for Alzheimer's Disease (CERAD); Digit Symbol Substitution Test (DSST).

[1]Calculated using binary logistic regression

Model 2 adjusted for age and gender

Model 3 adjusted for age and gender, race, educational level, marital status, income, BMI, energy, drinking status, smoking status, hypertension, diabetes, and stroke

*p <0.05; **p <0.01.

Table S4. Weighted odds ratios (95% confidence intervals) for scores on CERAD test, Animal fluency test and DSST test across quartiles of caffeine intake from coffee, stratified by gender, NHANES 2011-2014 (N =1803).

	**CERAD test**			Animal Fluency test			DSST		
	**Model 1[1]**	**Model 2[1]**	**Model 3[1]**	Model 1[1]	Model 2[1]	Model 3[1]	Model 1[1]	Model 2[1]	Model 3[1]
Women									
Caffeine (mg/day)									
<67	1.00 (Ref.)	1.00 (Ref.)	1.00 (Ref.)	1.00 (Ref.)	1.00 (Ref.)	1.00 (Ref.)	1.00 (Ref.)	1.00 (Ref.)	1.00 (Ref.)
67 to <124.5	0.63(0.34-1.14)	0.63(0.36-1.11)	0.70(0.39-1.27)	1.01(0.45-2.19)	1.16(0.58-2.45)	1.35(0.71-2.80)	0.83(0.47-1.48)	0.85(0.48-1.51)	0.87(0.38-2.01)
124.5 to <208	0.31(0.18-0.51) **	0.32(0.19-0.53) **	0.34(0.17-0.65) **	0.52(0.31-0.85) *	0.56(0.33-0.97) *	0.69 (0.40-1.22)	0.58(0.24-1.39)	0.59(0.26-1.31)	0.46(0.18-1.19)
≥208	0.55(0.30-0.99) *	0.59(0.33-1.05)	0.72(0.39-1.33)	1.14(0.53-2.44)	1.30(0.61-2.78)	1.56(0.75-3.26)	0.33(0.18-0.59) **	0.33(0.18-0.61) **	0.39(0.20-0.76) **
Men									
Caffeine (mg/day)									
<67	1.00 (Ref.)	1.00 (Ref.)	1.00 (Ref.)	1.00 (Ref.)	1.00 (Ref.)	1.00 (Ref.)	1.00 (Ref.)	1.00 (Ref.)	1.00 (Ref.)
67 to <124.5	0.79(0.44-1.44)	0.81(0.44-1.47)	0.92(0.46-1.81)	0.87(0.51-1.47)	0.89(0.53-1.46)	0.97(0.53-1.76)	0.65(0.34-1.21)	0.65(0.35-1.20)	0.74(0.37-1.50)
124.5 to <208	0.88(0.41-1.92)	0.87(0.39-1.97)	0.99(0.45-1.98)	0.84(0.48-1.46)	0.82(0.48-1.38)	1.05(0.58-1.89)	0.51(0.28-0.91) *	0.49(0.28-0.85) *	0.58(0.28-1.22)
≥208	0.59(0.34-0.99) *	0.61(0.35-1.06)	0.91(0.49-1.66)	0.49(0.30-0.81) *	0.53(0.32-0.87) *	0.71(0.37-1.33)	0.47(0.22-0.99) *	0.50(0.23-1.07)	0.87(0.35-2.17)

Abbreviations: The Consortium to Establish a Registry for Alzheimer's Disease (CERAD); Digit Symbol Substitution Test (DSST).

[1]Calculated using binary logistic regression

Model 2 adjusted for age

Model 3 adjusted for age, race, educational level, marital status, income, BMI, energy, drinking status, smoking status, hypertension, diabetes, and stroke

**p* <0.05; ***p* <0.01.

Table 5. Weighted Regression coefficients (β) and 95% confidence intervals for scores on CERAD test, Animal Fluency test, and DSST test across total coffee intake, NHANES 2011–2014 (N = 2513).

	Total coffee intake (g/day)
**CERAD test Score**	
Model 1[1]	0.0005(-0.0004-0.0016)
Model 2[1]	0.0005(-0.0003-0.0015)
Model 3[1]	-0.0001(-0.001-0.0009)
**Animal Fluency test Score**	
Model 1[1]	0.0010(0.0001-0.0018) *
Model 2[1]	0.0007(-0.0001-0.0014)
Model 3[1]	0.0003(-0.0004-0.0012)
**DSST test Score**	
Model 1[1]	0.0030(0.001-0.005) **
Model 2[1]	0.0029(0.0008-0.005) **
Model 3[1]	0.0017(0.0001-0.003) *

Abbreviations: The Consortium to Establish a Registry for Alzheimer's Disease (CERAD); Digit Symbol Substitution Test (DSST).

[1]Calculated using liner regression

Model 2 adjusted for age and gender

Model 3 adjusted for age and gender, race, educational level, marital status, income, BMI, energy, drinking status, smoking status, hypertension, diabetes, and stroke

***p <0.05; **p <0.01.**

Table 6. Weighted Regression coefficients (β) and 95% confidence intervals for scores on CERAD test, Animal Fluency test, and DSST test across caffeinated coffee and decaffeinated coffee, NHANES 2011–2014 (N = 2513).

	Caffeinated coffee (g/day)	Decaffeinated coffee (g/day)
**CERAD test Score**		
Model 1[1]	0.0005(-0.0005-0.0016)	0.0003(-0.001-0.0016)
Model 2[1]	0.0005(-0.0006-0.0016)	0.0004(-0.0009-0.0017)
Model 3[1]	0.00002(-0.001-0.0011)	0.0002(-0.0009-0.0014)
**Animal Fluency test Score**		
Model 1[1]	0.0012(0.0004-0.0019) **	-0.0006(-0.0017-0.0005)
Model 2[1]	0.0008(-0.0001-0.0016) *	-0.0004(-0.0016-0.0009)
Model 3[1]	0.0006(0.00001-0.0013) *	-0.0005(-0.0017-0.0007)
**DSST test Score**		
Model 1[1]	0.0027(0.0005-0.0049) *	0.0023(-0.0012-0.0058)
Model 2[1]	0.0025(0.0003-0.0046) *	0.0027(-0.0004-0.0059)
Model 3[1]	0.0021(0.0003-0.004) *	0.0022(-0.0005-0.005)

Abbreviations: The Consortium to Establish a Registry for Alzheimer's Disease (CERAD); Digit Symbol Substitution Test (DSST).

[1]Calculated using liner regression

Model 2 adjusted for age and gender

Model 3 adjusted for age and gender, race, educational level, marital status, income, BMI, energy, drinking status, smoking status, hypertension, diabetes, and stroke

***p <0.05; **p <0.01.**

Table 7. Weighted Regression coefficients (β) and 95% confidence intervals for scores on CERAD test, Animal Fluency test, and DSST test across caffeine intake from coffee, NHANES 2011–2014 (N = 1803).

	Caffeine intake (g/day)
**CERAD test Score**	
Model 1[1]	0.0044(0.0017-0.0071) **
Model 2[1]	0.0027(0.0001-0.0053) *
Model 3[1]	0.0025(0.0001-0.0049) *
**Animal Fluency test Score**	
Model 1[1]	0.0018(-0.0010-0.0047)
Model 2[1]	0.0011(-0.0018-0.0039)
Model 3[1]	-0.0002(-0.0028-0.0023)
**DSST test Score**	
Model 1[1]	0.0073(0.0001-0.0145) *
Model 2[1]	0.0049(-0.0016-0.011)
Model 3[1]	0.0046(-0.0015-0.010)

Abbreviations: The Consortium to Establish a Registry for Alzheimer's Disease (CERAD); Digit Symbol Substitution Test (DSST).

[1]Calculated using liner regression

Model 2 adjusted for age and gender

Model 3 adjusted for age and gender, race, educational level, marital status, income, BMI, energy, drinking status, smoking status, hypertension, diabetes, and stroke

$*p < 0.05$; $**p < 0.01$.
